# Silver As Antibacterial toward *Listeria monocytogenes*

**DOI:** 10.3389/fmicb.2016.00307

**Published:** 2016-03-07

**Authors:** Simone Belluco, Carmen Losasso, Ilaria Patuzzi, Laura Rigo, Daniele Conficoni, Federica Gallocchio, Veronica Cibin, Paolo Catellani, Severino Segato, Antonia Ricci

**Affiliations:** ^1^Department of Food Safety, Istituto Zooprofilattico Sperimentale delle VenezieLegnaro, Italy; ^2^Department of Animal Medicine, Production and Health, Università di PadovaPadova, Italy; ^3^Department of Information Engineering, Università di PadovaPadova, Italy

**Keywords:** food safety, nanoparticles, food preservation, cross-contamination, foodborne pathogens

## Abstract

*Listeria monocytogenes* is a serious foodborne pathogen that can contaminate food during processing and can grow during food shelf-life. New types of safe and effective food contact materials embedding antimicrobial agents, like silver, can play an important role in the food industry. The present work aimed at evaluating the *in vitro* growth kinetics of different strains of *L. monocytogenes* in the presence of silver, both in its ionic and nano form. The antimicrobial effect was determined by assaying the number of culturable bacterial cells, which formed colonies after incubation in the presence of silver nanoparticles (AgNPs) or silver nitrate (AgNO_3_). Ionic release experiments were performed in parallel. A different reduction of bacterial viability between silver ionic and nano forms was observed, with a time delayed effect exerted by AgNPs. An association between antimicrobial activity and ions concentration was shown by both silver chemical forms, suggesting the major role of ions in the antimicrobial mode of action.

## Introduction

*Listeria monocytogenes* is a foodborne pathogen responsible for severe disease in immunocompromised people and for non-invasive infections in immunocompetent individuals (Crum, 2002). Its incidence has been estimated as 0.41 and 0.26 cases per 100,000 inhabitants in Europe in 2012 (EFSA and ECDC, 2014) and in the US in 2013 (CDC, 2014), respectively. Despite the low incidence rate, *L. monocytogenes* is ranked among the top-most serious foodborne pathogens due to its high pathogenicity, as is shown by methods for the quantification of disease burden, such as DALY/100,000 cases (Havelaar et al., 2012). Due to its widespread presence in the environment, *L. monocytogenes* has a high potential to contaminate food during processing and distribution (Latorre et al., 2007). Ready-to-eat foods (RTE) pose a main risk for human listeriosis infection due to the lack of effective decontamination treatment (e.g., heat), which otherwise would inactivate viable *L.* *monocytogenes* cells (Parisi et al., 2010; Lomonaco et al., 2011), or due to the potential for recontamination after applied treatment.

The Scientific Committee on Veterinary Measures relating to Public Health (SCVPH) has recommended, as an objective for public health, that the level of *L. monocytogenes* in food be below 100 cfu/g (Reg. 2073/2005). This safety criterion has been set in relevant EU regulations for RTE foods during their entire shelf life. US food policy on *L. monocytogenes* is characterized by more stringent requirements regarding the monitoring process in processing plants, in order to avoid food contamination (FSIS, 2014).

Nevertheless, the ability of *Listeria* spp. to grow between 0 and 4°C and its tolerance to some commonly applied preservatives, like salt, explain the challenge of applying alternative control strategies along the whole food chain (Latorre et al., 2007).

The shelf life for such products is often calculated based on the kinetics of bacterial growth. Temperature control is critical in the overall control of *L. monocytogenes* in RTE foods. However, lack of proper control at commercial (production, transport, retail) or consumer levels can result in failure to control growth of the pathogen.

In this context, the development of new types of safe and effective food contact materials able to extend the shelf life of food products or to prevent cross contamination is of great interest. Among different chemicals with antimicrobial activity, silver is considered as a good candidate, as it is known to exert antimicrobial properties toward both Gram positive and Gram negative bacteria (Silver, 2003; Rai et al., 2009; Bae et al., 2010; Fayaz et al., 2010; You et al., 2011; Maillard and Hartemann, 2013).

Previous studies have shown that nanoparticles (NPs) could be used as effective bactericidal materials in antimicrobial formulations due to their enhanced reactivity, resulting from their high surface/volume ratio (Pal et al., 2007; Choi et al., 2008; Park et al., 2009). In particular, silver in NP form (AgNPs) is known to exhibit strong biocidal effects on different bacterial species (Sondi and Salopek-Sondi, 2004; Rai et al., 2009; Losasso et al., 2014; Berton et al., 2015), including multidrug resistant bacteria (Lara et al., 2010). However, it has been demonstrated that not only different bacterial species but also different strains within one species can show different sensitivities to AgNPs (Losasso et al., 2014). The advantage of using AgNPs has been investigated under real life scenarios to increase fruit shelf life (Gudadhe et al., 2014) and to reduce the contamination by coliforms in cheese (Beigmohammadi et al., 2016). However, to our knowledge the antimicrobial effect of AgNPs against *L. monocytogenes* has never been proved.

Nevertheless, data arising from studies on the effectiveness of NPs as antimicrobials are difficult to interpret due to the specific chemical and physical characteristics of these particles. Thus, it is of primary importance to provide detailed characterization of any tested NPs starting from size and shape evaluation, as this would allow the scientific community to compare the results.

The aims of this work were to evaluate the efficacy of AgNPs as an antimicrobial toward *L. monocytogenes* and to explore intra-species variability and its relation with antimicrobial resistance. Moreover, the effectiveness of AgNPs as an antimicrobial material is discussed in the light of its mechanism of action.

## Materials and Methods

### Nanoparticles and Chemicals

Silver nanoparticles colloidal suspension in citrate 2 mM (20 nm Citrate BioPure™ Silver, batch numbers: MGM2167 e KJW1896) and their control dispersant medium were purchased from Nanocomposix (San Diego, CA, USA). Silver nitrate (AgNO_3_) 1000 mg L^–1^ stock solution was purchased from Ultra Scientific (North Kingstown, RI, USA).

### *L. monocytogenes* Isolates

Twenty *L. monocytogenes* wild isolates, originating from different food matrices, were obtained from the collection of pathogenic microorganisms of the Food Microbiology laboratory of the Istituto Zooprofilattico Sperimentale delle Venezie (Legnaro, Italy); details are presented in **Table 1**. *L. monocytogenes* ATCC^®^ 13932^TM^ was also examined. The isolates were stored in Microbank™ (Pro-Lab Diagnostics, US) at -80°C until needed. All strains were identified as *L. monocytogenes* according to ISO standard (ISO, 1998).

**Table 1:** *Listeria monocytogenes* isolates investigated.

Isolate number	Serotype	Isolation year	Food source
ATCC			^–^
1	1/2c	2013	Salami (Pork)
2	1/2c	2013	Hamburger (Beef)
3	1/2a	2013	Poultry meat with pepper to be cooked on a spit
4	1/2a	2013	Horse
5	1/2c	2013	Home-made salami
6	1/2a	2013	Meat balls (Beef and Turkey)
7	4b	2013	Pork sausage
8	1/2a	2013	Smoked salmon
9	1/2c	2013	Piadina with sausage
10	4c	2014	Soft cheese (Goat-cow)
11	1/2a	2013	Smoked trout
12	4b	2013	Soft cheese (Cow)
13	1/2c	2013	Poultry roll
14	3c	2013	Sausage (Pork-Beef)
15	1/2b	2013	Roasted turkey
16	1/2a	2013	Smoked salmon
17	1/2b	2013	Pork
18	1/2a	2013	Hamburger
19	1/2a	2012	Alpine hut butter
20	1/2a	2012	Soft cheese (Ewe)

### Size and Morphology of AgNPs

Morphological parameters of AgNPs were verified using a transmission electron microscope (TEM) JEM 2000 EX II (JEOL^®^) (120 KV) at the Department of Technology and Management of Industrial Systems (University of Padua). AgNPs were diluted with Muller Hinton Broth (MHB) culture media to reach a final concentration of 50 mg/L. TEM observations were performed at 6000X and 100KX and microphotographs were elaborated using ImageJ^®^ software, enabling evaluation of average particle diameter and roundness for a sample of 500 particles.

### Antibiotics Susceptibility Testing

*Listeria monocytogenes* isolates were tested for susceptibility to antimicrobials (detailed in Supplementary Table 1), using a commercial microdilution test (Sensititre^®^ Streptococcus panel ITST4F) according to the manufacturer’s recommendations. The results were visually evaluated after 48 h of incubation at 37°C and the minimum inhibitory concentration (MIC) was defined as the lowest concentration able to completely inhibit bacterial growth.

### Silver Susceptibility Assay

In this experiment a two steps susceptibility assay was performed.

#### First Step

The different susceptibility of the 21 *L. monocytogenes* isolates to different concentrations of AgNPs and AgNO_3_ was assessed as follows. *L. monocytogenes* isolates from Microbank™ were subcultured in Tryptone Soya supplemented with yeast and incubated at 37°C overnight. The day after a loopful was transferred to 15 ml of Mueller Hinton Broth (MHB) and incubated at 37°C overnight to produce the inocula. MHB was used as culture medium with the objective of obtaining a good compromise between *L. monocytogenes* growth and NPs stability, given that this is a quite simple and non-selective liquid medium, specifically designed for susceptibility studies and having the minimum requirements for bacteria growth (Gilbert et al., 1987). The final liquid culture in MHB consisted of *L. monocytogenes* in the Lag phase (OD A_600_ = 0.3 = 10^8^ CFU/ml) (vlab.amrita.edu, 2011; CLSI, 2012) in the presence of different concentration of AgNPs (100, 50 mg/L^–1^) or AgNO_3_ (60, 30, 5 mg/L^–1^). The tubes were incubated in an orbital shaker at 200 rpm at 37°C for 4 h. A positive control (flask containing AgNPs and MHB) and a negative control (flask containing inoculum and MHB) were included. The high rotary shaking speed was selected to prevent the aggregation and settlement of the NPs over the incubation period and Ag concentration levels were selected according to previous observations with *Salmonella* (Losasso et al., 2014).

After 4 h of incubation, a volume of 50 μl was removed from liquid culture and, after serial 10-fold dilutions in MHB broth up to 10^–8^ of *L. monocytogenes*, 100 μl aliquots were spread-plated on ALOA Agar according to Ottaviani et al. (1997). Colonies were counted after incubation of spread-plates at 37°C for 48 h, and counts were expressed as log CFU/ml.

#### Second Step

The time dependent evaluation of the effectiveness of silver as an antilisterial on selected *L. monocytogenes* isolates was evaluated. A cell culturability assay was carried out as described above, with different concentrations of AgNPs (300 mg/L^–1)^ and AgNO_3_ (30, 20, and 10 mg/L^–1^) at different time points (1, 4, 24, 48, and 72 h). Each experiment was performed in triplicate. Positive and negative controls were included.

### Ionic Release

*Listeria monocytogenes* (strain 5) liquid culture were prepared as in the antimicrobial susceptibility assay and incubated in the presence of AgNPs (300 ppm) and AgNO_3_ (30 ppm). After 0, 24, 48, and 72 h of incubation in an orbital shaker (200 rpm, 37°C), 250 μl of each suspension was transferred to a centrifuge tube (Microcon-30 kDa Centrifugal Filter Unit with Ultracel-30 membrane – Merckmillipore, Ltd) and centrifuged at 5000 *g* for 60 min. After filtration, filtered samples were diluted with milli-Q water in the ratio of 1:500 as preparation for atomic absorption analysis.

Total silver was quantified in suitably diluted samples by Electrothermal Atomic Absorption Spectrometry (ETAAS) using an M6 mkII Atomic Absorption Spectrometer (Thermo Electron, Cambridge, UK) with D2 and Zeeman background correction, equipped with a GF95 Graphite Furnace atomiser (GFAAS). Analytical conditions are reported in Supplementary Tables 2 and 3. Calibration solutions for ETAAS were prepared within the range of 0.3–3 μg L^–1^ (corresponding to 150–1500 μg L^–1^ in samples) by dilution of the AgNO_3_ stock solution with Milli-Q water. Data acquisition and processing was conducted using thermo solaar software to evaluate the recovery of ionic silver after filtration, and thus its potential interaction with the centrifuge tube filter, an additional parallel extraction was performed. Briefly, three solutions of 30 ppm of AgNO_3_ were prepared in MHB without cells. The ion concentrations were measured before and after filtration to obtain a recovery factor applicable to all ionic release data.

For ionic release assessment, two values per time point (24, 48, and 72 h) were acquired and corrected by a recovery factor, obtained by calculating the percentage ratio between the total amount of silver ions released after AgNO_3_ dilution (before centrifugation) and the amount of total filtered silver (after centrifugation). A mean value was calculated.

### Data Analysis

*C*ounts resulting from AgNPs susceptibility assay were log-transformed and then summarized as mean values and standard errors. For statistical purposes a value of 300 colonies was assigned to plates on which more than 300 colonies were observed according to ISO 7218/2007 (ISO, 2007).

A *t*-test was used to determine whether the differences between strains that arose from graphical analysis were also noteworthy at statistical level. A classical 5% threshold for significance was used to detect inter strains differences at different time points (*p*-value <0.05). A Wilcoxon test was also performed to verify the accordance to the *t*-test.

## Results

### AgNPs Characterization

**FIGURE 1:**
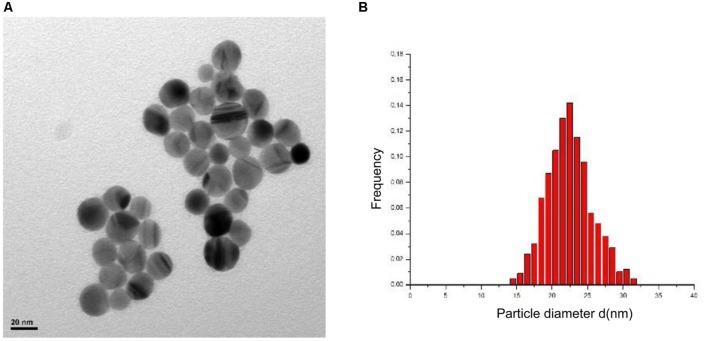
**Results of silver nanoparticles (AgNPs) characterization with TEM.** Morphology **(A)** and dimensional distribution **(B)**.

As visualized by TEM, AgNPs suspension appeared as a mixture of homogeneous particles by size and shape (**Figure 1A**). Most particles were spherical, but the sporadic presence of regular polygonal particles was also observed. Particle size distribution showed one major population, with median diameters of 23(±2) nm (**Figure 1B**).

### Antimicrobial Susceptibility Testing

The results of the phenotypic antimicrobial susceptibility test of the 21 *L. monocytogenes* isolates are reported in Supplementary Table 1. No relevant differences among strains were observed.

### Silver Susceptibility Assay

Differences in the susceptibility of *L. monocytogenes* strains were evaluated trough a two steps experiments. The first step resulted in the selection of six strains 4,5,7,11,20 according to their susceptibility and ATCC, with strains 4,5,11 being the less susceptible and 7, 20, and ATCC being the more susceptible.

**FIGURE 2:**
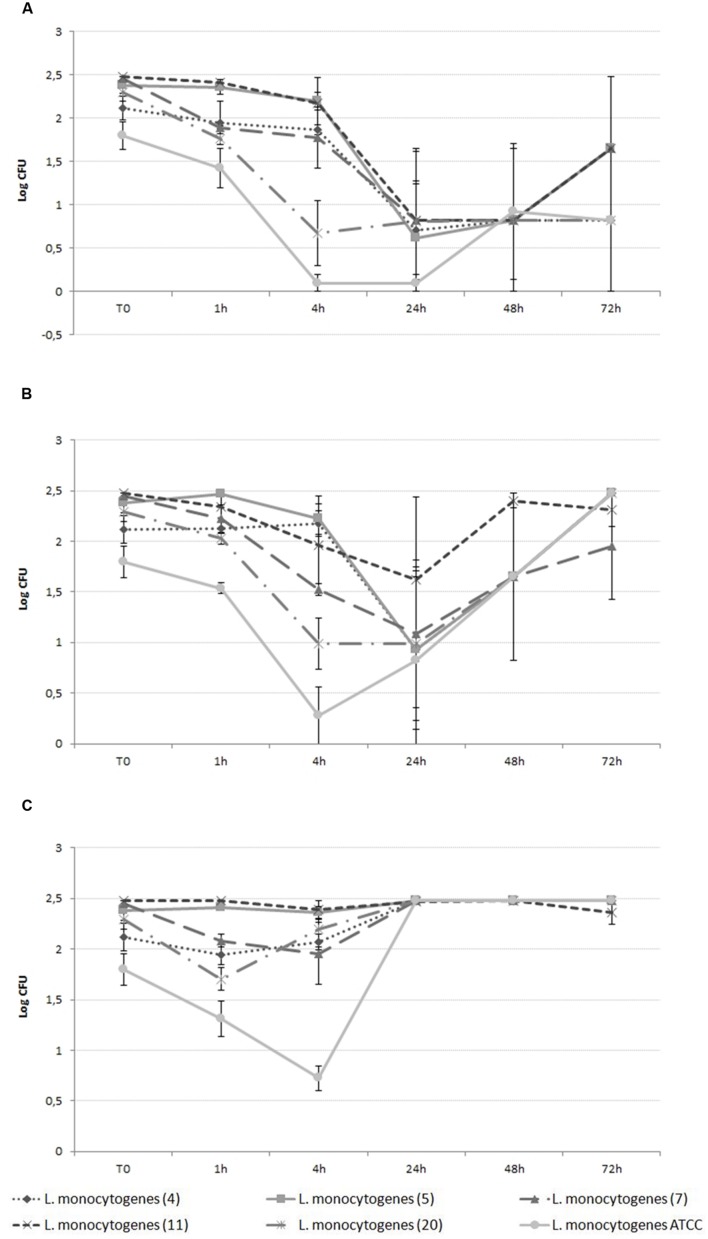
**Growth curve of *L. monocytogenes* in the presence of 30 ppm (A), 20 ppm (B), and 10 ppm (C) AgNO_3_**.

**FIGURE 3:**
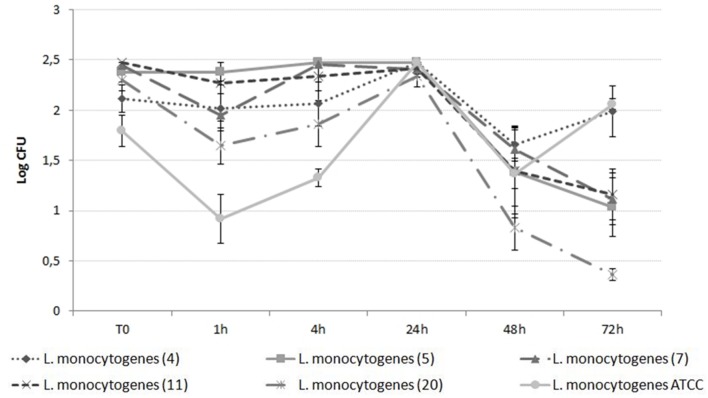
**Growth curve of *L. monocytogenes* in the presence of 300 ppm AgNPs**.

Results of the second step are shown in **Figures 2** and **3**. *L. monocytogenes* counts were reduced to differing extents depending on the antimicrobial agent, with AgNO_3_ being effective at lower doses from the beginning of incubation (**Figure 2**) and AgNPs showing effectiveness only after 24 h incubation (**Figure 3**). The effect caused by AgNO_3_ was proportional to the dose, with 30 mg/L being the most effective, and 10 mg/L the least effective for all six *L. monocytogenes* isolates (**Figure 2**). Some statistically significant differences were observed among the selected strains at different time points. The maximum difference among strains was observed after 4 h of incubation both in the presence of AgNO_3_ and AgNPs. Conversely, no differences were detected after 24 h of incubation in the presence of AgNO_3_. In detail, in the presence of AgNO_3_ (30 and 20 ppm) *L. monocytogenes* ATCC and strain 20 counts were lower than the others (*p* < 0.05). ATCC strain exerted statistically significant lower counts also when incubated with 10 ppm of AgNO_3_ difference is confirmed in the case of ATCC. In the case of AgNPs a behavior similar to AgNO_3_ (30 ppm) could be described with statistically significant differences at 4h (ATCC and strain 20 more susceptible) and a convergence of growth curves at 24 h. In this case, however, differences among strains were also observed after 48 h with strain 20 being more sensitive than strains 4 and 7 (*p* < 0.05). Interestingly after 72 h ATCC, as also strain 4, showed a statistically significant recovery if compared with other *L. monocytogenes* strains.

### Ionic Release

**FIGURE 4:**
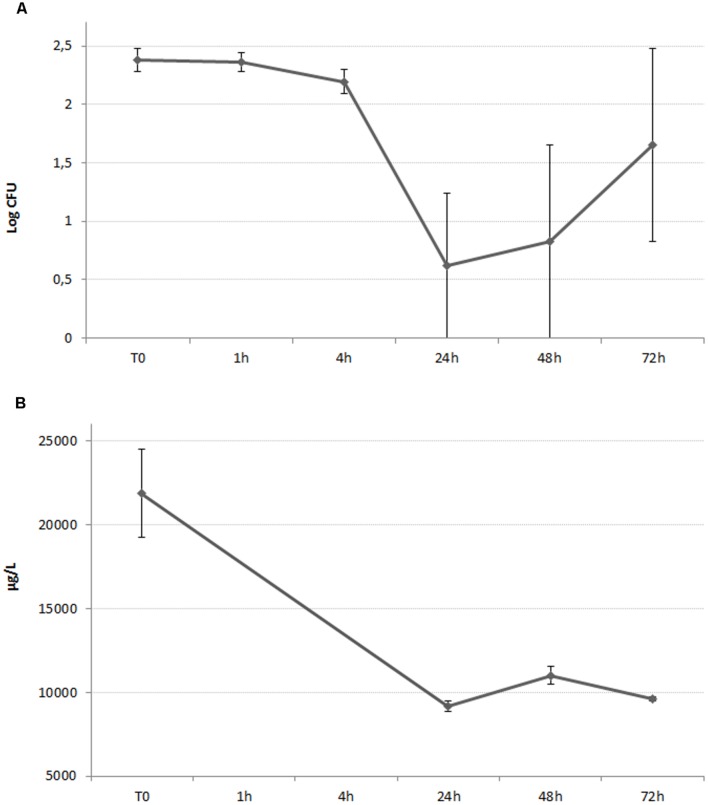
**(A)** Growth curve of *L. monocytogenes* in the presence of AgNO_3_, 30 ppm. The graphed values represents the mean of the counts belonging to *Listeria monocytogenes* strain n°5. **(B)** Time course assay of the silver ionic release of a 30 ppm solution of AgNO_3_, incubated in the presence of *L. monocytogenes* isolate 5.

**FIGURE 5:**
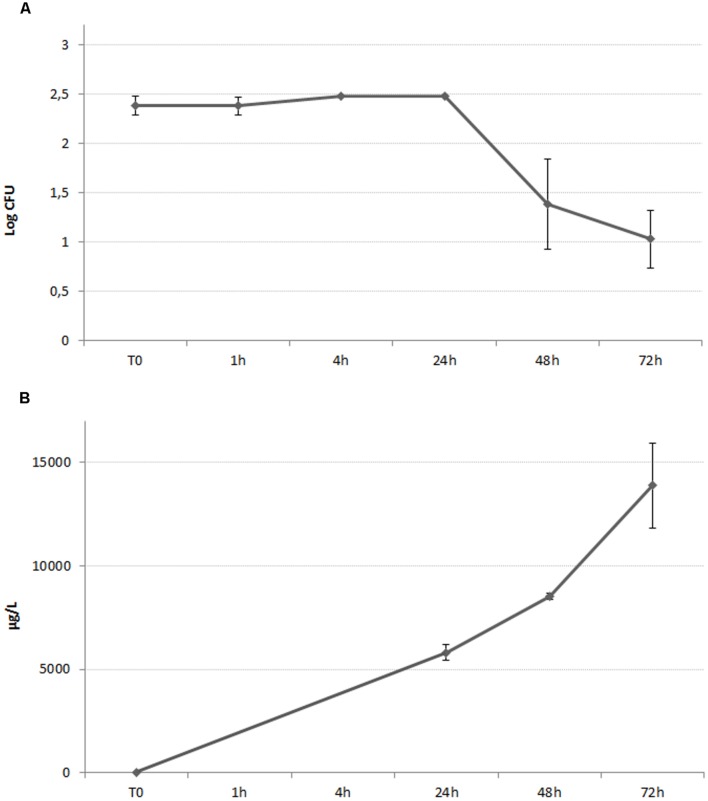
**(A)** Growth curve of *L. monocytogenes* in the presence of AgNPs 300 ppm. The graphed values represents the mean of the counts belonging to *L. monocytogenes* strain n°5. **(B)** Time course assay of the silver ionic release of a 300 ppm suspension of AgNPs, incubated in the presence of *L. monocytogenes* isolate 5.

Some correlation between ionic release and strain 5 growth curve was found both in the case of AgNO_3_ (**Figure 4**) and AgNPs (**Figure 5**), even though the relative extents were different. As shown in **Figure 4B**, in the case of AgNO_3_ a large amount of released silver ions was detected in the analyzed solution at the beginning of the study, when the measured silver concentration was approximately 22,000 μg/L. At this concentration, AgNO_3_ was able to immediately exert the expected antilisterial activity (**Figure 4A**).

The amount of silver ions released from AgNPs increased over time, with a maximum observed concentration of approximately 14,000 μg/L after 72 h of incubation (**Figure 5B**). As suggested by **Figure 5A**, the antilisterial effect exerted by AgNPs started after 24 h of incubation, and after the released silver ions reached the concentration of approximately 6,000 μg/L.

## Discussion

This study aimed to investigate the effectiveness of silver in ionic and nano form as antimicrobial toward a panel of *L. monocytogenes* isolates.

The results obtained showed that both chemical forms of silver exerted antimicrobial activity and should, therefore, be considered as suitable to be used as biocide against *L. monocytogenes*. However, the timing of the inhibitory effect was different as AgNPs exhibited time-delayed inhibition of *L. monocytogenes*, compared with AgNO_3_.

The antimicrobial activity of AgNPs has been commonly described in the literature, and a wide array of microbes, including drug-resistant bacteria, fungi, and viruses, have been examined (Sondi and Salopek-Sondi, 2004; Pal et al., 2007; Choi et al., 2008; Fayaz et al., 2010; Lara et al., 2010; Samberg et al., 2011; You et al., 2011). To our knowledge, however, delayed effects of AgNPs on bacterial inhibition have never been observed. The delayed effect of AgNPs on the pathogen may have been caused by the gradual release of Ag ions from the AgNPs.

Our previous study demonstrated that the effectiveness of AgNPs as an antimicrobial toward *Salmonella enterica* was strongly strain-dependent due to the expression of silver specific resistance determinant by the resistant strains (Losasso et al., 2014). In the present study, despite the selection of *L. monocytogenes* isolates originating from different food matrices, the inclusion of an ATCC strain and the initial screening to select strains showing different behaviors toward silver, no relevant differences in Ag susceptibility assays were detected, and no differences among the examined isolates were noted.

In our study toxicity and dissolution tests were performed in parallel, as silver ions released upon dissolution of AgNPs have been proposed to be the primary contributor to the antibacterial activity of these compounds (Silver, 2003). The relation between the sensitivity assay and the amount of ions at the tested time points, in both cases (AgNPs and AgNO_3_) suggests that ions are the more effective elements exerting antibacterial activity. This findings agrees with scientific literature where several studies demonstrates that cations could interfere with the normal function of the bacterial electron transport chain, and thus, be responsible for ROS (Reactive Oxygen Species) formation at the cell membrane (Park et al., 2009; Xiu et al., 2012; Agnihotri et al., 2013). However, another study, using gene deletion strains, revealed that different pathways could be involved in the bacterial response to silver stress, as NPs can also exert a direct toxic effect (Ivask et al., 2014). Both ion-mediated toxicity as well as the physical interaction between NPs and the bacterial wall appear to contribute to the inhibitory effect, thus physicochemical properties of the NPs examined and the media used for NP suspension might play an important role (Prabhu and Poulose, 2012; Agnihotri et al., 2013).

## Conclusion

Our study suggests that *L. monocytogenes* is sensitive to silver and that the efficacy is linked to ionic release. We speculate that silver-based food contact materials could play a useful role in the food industry, perhaps to reduce surface contamination, or eventually, to prolong shelf life. However, to confirm this, studies under industrial conditions are needed, to evaluate silver efficacy as well as the potential, hazardous migration of silver from food contact materials into food (Pezzuto et al., 2015). As persistent nanomaterials, like silver, raise some concerns in terms of potential migration and consequent ingestion by consumers, its inclusion in food contact materials should take into account the predicted use to avoid such drawbacks. In this context the development of nanocomposite materials embedding silver is of particular interest (Abreu et al., 2015). It is important to emphasize that the present study took into consideration only one type of AgNP; the current results cannot be generalized due to the broad variability of AgNPs on biological activity, which depends, not only on their chemical forms, but also on their specific shapes and dimensions (Gogoi et al., 2006; Xiu et al., 2012).

## Author Contributions

SB, CL, AR conceived and designed the research, acquired and interpreted the data, drafted the work; IP analyzed the data; LR, DC, FG acquired the data; VC, PC, SS conceived the research and interpreted the data.

## Conflict of Interest Statement

The authors declare that the research was conducted in the absence of any commercial or financial relationships that could be construed as a potential conflict of interest.
