# Supplementary material for: Silver As Antibacterial toward Listeria monocytogenes
Source: Front Microbiol. 2016 Mar 7;7:307. doi: 10.3389/fmicb.2016.00307 (PMC4779933; doi:10.3389/fmicb.2016.00307)
Supplement: Supplementary file 1 [file Table_S1.DOCX]

Table S1: Antimicrobials susceptibility values. The acronyms indicates the following antibiotic molecules (AMP: ampicillin; FOT: cefotaxim; OXA: Oxacillin; CLI: Clindamycin; DAP: Daptomycin; DOX: Doxycycline; ERY: Erytromycin; LEVO: Levofloxacin; LZD: Linezolid; MERO: Meropenem; MXF:Moxifloxacin; PEN: Penicillin; TET: tetracycline; SXT: co-trimoxazole; VAN:Vancomycin)

| **MIC (µg/ml)** | | **AMP** | **FOT** | **OXA** | **CLI** | **DAP** | **DOX** | **ERY** | **LEVO** | **LZD** | **MERO** | **MXF** | **PEN** | **TET** | **SXT** | **VAN** |
| --- | --- | --- | --- | --- | --- | --- | --- | --- | --- | --- | --- | --- | --- | --- | --- | --- |
| **13932** | **ATCC** | 0,5 | >4 | >4 | >0,5 | >1 | 0.25 | 0,25 | 1 | 2 | 0,12 | >1 | 0,5 | ≤0.25 | ≤0.12 | 1 |
| **5408** | **1** | 0,25 | >4 | >4 | >0,5 | >1 | ≤0.12 | 0,25 | 1 | 2 | 0,06 | >1 | 0,12 | ≤0.25 | ≤0.12 | 1 |
| **5378** | **2** | 0,25 | >4 | >4 | >0,5 | >1 | ≤0.12 | 0,25 | 1 | 2 | 0,12 | 0,5 | 0,25 | ≤0.25 | ≤0.12 | 1 |
| **5739** | **3** | 0,25 | >4 | >4 | >0,5 | >1 | 0.25 | 0,25 | 1 | 2 | 0,12 | 0,5 | 0,25 | ≤0.25 | ≤0.12 | 1 |
| **5807** | **4** | 0,25 | >4 | >4 | >0,5 | >1 | ≤0.12 | 0,25 | 1 | 2 | 0,06 | 0,5 | 0,12 | ≤0.25 | ≤0.12 | 1 |
| **6258/1** | **5** | 0,25 | >4 | >4 | >0,5 | >1 | 0.25 | 0,25 | 1 | 2 | 0,12 | 0,5 | 0,25 | ≤0.25 | ≤0.12 | 1 |
| **6334/1** | **6** | 0,25 | >4 | >4 | >0,5 | >1 | ≤0.12 | 0,25 | 1 | 2 | 0,06 | 0,5 | 0,12 | ≤0.25 | ≤0.12 | 1 |
| **7047/1** | **7** | 0,5 | >4 | >4 | >0,5 | >1 | 0.25 | 0,25 | 1 | 2 | 0,12 | 0,5 | 0,5 | ≤0.25 | ≤0.12 | 1 |
| **6840/5** | **8** | 0,25 | >4 | >4 | >0,5 | >1 | 0.25 | 0,25 | 1 | 2 | 0,06 | 0,25 | 0,25 | ≤0.25 | ≤0.12 | 1 |
| **7001/2** | **9** | 0,25 | >4 | >4 | >0,5 | >1 | ≤ 0.12 | 0,25 | 1 | 2 | 0,12 | 0,5 | 0,25 | ≤0.25 | ≤0.12 | 1 |
| **125** | **10** | 0,5 | >4 | >4 | >0,5 | >1 | 0.25 | 0,25 | 1 | 2 | 0,25 | 0,5 | 0,5 | ≤0.25 | ≤0.12 | 1 |
| **2447** | **11** | 0,25 | >4 | >4 | >0,5 | >1 | 0.25 | 0,25 | 1 | 2 | 0,12 | 0,5 | 0,25 | ≤0.25 | ≤0.12 | 1 |
| **2301/1** | **12** | 0,5 | >4 | >4 | >0,5 | >1 | 0.25 | 0,25 | 1 | 2 | 0,12 | 0,5 | 0,5 | ≤0.25 | ≤0.12 | 1 |
| **2827/3** | **13** | 0,25 | >4 | >4 | >0,5 | >1 | >2 | 0,25 | 1 | 2 | 0,06 | 0,5 | 0,25 | ≤0.25 | ≤0.12 | 1 |
| **2501/2** | **14** | 0,25 | >4 | >4 | >0,5 | >1 | 0.25 | 0,25 | 1 | 2 | 0,12 | 0,5 | 0,25 | ≤0.25 | ≤0.12 | 1 |
| **2528/4** | **15** | 0,12 | >4 | >4 | >0,5 | >1 | ≤ 0.12 | 0,12 | 0,5 | 2 | 0,06 | 0,25 | 0,25 | ≤0.25 | ≤0.12 | 1 |
| **3340** | **16** | 0,25 | >4 | >4 | >0,5 | >1 | ≤ 0.12 | 0,12 | 1 | 2 | 0,06 | 0,5 | 0,25 | ≤0.25 | ≤0.12 | 0,5 |
| **4333/1** | **17** | 0,25 | >4 | >4 | >0,5 | >1 | 0.25 | 0,25 | 1 | 2 | 0,12 | 0,25 | 0,25 | ≤0.25 | ≤0.12 | 1 |
| **3663/1** | **18** | 0,25 | >4 | >4 | >0,5 | >1 | ≤ 0.12 | 0,25 | 1 | 2 | 0,12 | 0,5 | 0,25 | ≤0.25 | ≤0.12 | 1 |
| **3908** | **19** | 0,25 | >4 | >4 | >0,5 | >1 | ≤ 0.12 | 0,25 | 1 | 2 | 0,12 | 0,25 | 0,25 | ≤0.25 | ≤0.12 | 1 |
| **5162** | **20** | 0,25 | >4 | >4 | >0,5 | >1 | 0.25 | 0,25 | 2 | 2 | 0,06 | 0,5 | 0,25 | ≤0.25 | ≤0.12 | 1 |
