# Supplementary material for: Silver As Antibacterial toward Listeria monocytogenes
Source: Front Microbiol. 2016 Mar 7;7:307. doi: 10.3389/fmicb.2016.00307 (PMC4779933; doi:10.3389/fmicb.2016.00307)
Supplement: Supplementary file 2 [file Table_S2.DOCX]

Table S2. Instrumental parameters for GFAAS determination

| **Parameter** | **Ag** |
| --- | --- |
| Wavelength (nm) | 328.1 |
| Slit (nm) | 0.5 |
| Measurement time (sec) | 3.0 |
| Background correction | D_2_ |
| Atomisation (t °C) | 1250 |
