# Supplementary material for: Silver As Antibacterial toward Listeria monocytogenes
Source: Front Microbiol. 2016 Mar 7;7:307. doi: 10.3389/fmicb.2016.00307 (PMC4779933; doi:10.3389/fmicb.2016.00307)
Supplement: Supplementary file 3 [file Table_S3.DOCX]

Table S3. GFAAS temperature program

| Step n° | Temp (°C) | Time (sec) | Ramp (°C sec^-1^) | Ar flow (L min^-1^) |
| --- | --- | --- | --- | --- |
| 1 | 110 | 20 | 5 | 0.1 |
| 2 | 160 | 10 | 5 | 0.1 |
| 3 | 550 | 30 | 30 | 0.2 |
| 4 | 1250 | 3 | 0 | Off |
| 5 | 2500 | 3 | 0 | 0.3 |
